# Serum lipid profiles and dyslipidaemia are associated with retinal microvascular changes in children and adolescents

**DOI:** 10.1038/srep44874

**Published:** 2017-03-20

**Authors:** Wei Xiao, Xinxing Guo, Xiaohu Ding, Mingguang He

**Affiliations:** 1 State Key Laboratory of Ophthalmology, Zhongshan Ophthalmic Center, Sun Yat-sen University, Guangzhou, China; 2 Centre for Eye Research Australia, University of Melbourne, Australia

## Abstract

The present study aims to assess the relationship between serum lipid parameters and retinal microvascular calibres in children and adolescents. A total of 950 participants aged 7 to 19 years were recruited. Central retinal arteriolar equivalent (CRAE) and central retinal venular equivalent (CRVE) were measured from digital retinal images. Serological testing was performed to obtain lipid profiles. Dyslipidaemia was defined according to the US national expert panel guideline. After adjusted for age, sex, mean arterial blood pressure, axial length, body mass index and the fellow retinal vascular calibre, no significant association was found between retinal vascular diameters and any lipid parameters (all *P* > 0.05) in children younger than 12 years. Among the adolescents 12 years and older, increased triglycerides, total cholesterol, low-density lipoprotein cholesterol, and apoB were associated with decrease in CRAE (*β* = −1.33, −1.83, −1.92 and −7.18, *P* = 0.031, 0.003, 0.006, and 0.009, respectively). Compared with normolipidemic counterparts, adolescents with dyslipidaemia had significantly narrower retinal arteriolar diameters. No significant relationship between lipid subclass levels and CRVE was revealed in adolescents. The present findings suggest that the elevation of atherogenic lipids in adolescents is closely related to the adverse changes of retinal arterioles. Dyslipidaemia may affect systemic microvasculature from childhood on.

Atherosclerosis, including ischemic heart disease, ischemic stroke, and peripheral arterial disease, is the most common cause of mortality and long-term disability worldwide^1^. Although manifest disease is rare in early-lifetime, precursor risk factors that potentially accelerate the onset of atherosclerosis can present from childhood on. Among the known cardio-metabolic risk factors, dyslipidaemia is one of the most predominant but modifiable factors. For example, serum levels of total cholesterol (TC), low-density lipoprotein cholesterol (LDL-C), and triglycerides (TG) in children are each predictors of coronary artery calcium and increased carotid intima-media thickness (cIMT), both of which are precursors of advanced atherosclerosis^2^,^3^,^4^. In contrast, children with familial hypercholesterolaemia had smaller increase in cIMT when they accepted lipid lowering therapy^5^. Based on such evidence, the Expert Panel on Integrated Guidelines for Cardiovascular Health and Risk Reduction in Children and Adolescents was released by the National Heart, Lung, and Blood Institute (NHLBI) in 2011^6^. The Panel emphasized that early identification and management of dyslipidaemia throughout youth and into adulthood would be beneficial to reduce the risk of clinical cardiovascular disease in adult life.

Substantial epidemiologic evidence has revealed that cardiovascular disease (CVD) and its risk factors are tightly related to microcirculatory changes^7^,^8^. Due to the shared anatomical and physiological similarities between the retinal, cerebral and myocardial microvasculature, retinal vessels is considered as a favorable surrogate for the systemic microvascular system. By using digital retinal photography and computer-aided analysis technique, many subtle but quantifiable signs, typically the retinal vascular calibre, were found to be associated with various systemic, environmental and genetic factors, such as body mass index (BMI)^9^, body fat deposition^10^, and inflammatory markers^11^. As for serum lipid indices, the Blue Mountains Eye Study^12^ showed that elevated HDL-C was associated with narrower retinal arterioles and venules among people older than 49 years. In a clinical trial, patients with hypercholesterolemia showed widening of both retinal arterioles and venules after LDL-C apheresis, indicating an improvement of microcirculation perfusion^13^. Despite the sporadic but inconsistent results in adults, no literature exists reporting the association between lipid profiles and retinal vascular calibre specifically in children and adolescents. We hypothesized that lipid profiles would relate to retinal vascular diameters in youth. Using data from the Guangzhou Twin Eye Study, we tested our assumption among individuals aged 7 to 19 years old.

## Results

Demographic and clinical characteristics of the population are shown in Table 1. The present study included 438 children (214 boys and 224 girls) aged 7 to 11 years old, and 512 adolescents (239 boys and 273 girls) aged 12 years and older. Among the children, there were significant differences in LDL-C, apoA1, apoA1/apoB and axial length between boys and girls. Compared with young girls, boys had significantly lower levels of LDL-C (2.22 ± 0.61 mmol/L vs. 2.36 ± 0.59 mmol/L, *P* = 0.022), but higher apoA1 (1.43 ± 0.21 g/L vs. 1.38 ± 0.18 g/L, *P* = 0.021) and greater axial length (23.7 ± 1.01 mm vs. 23.2 ± 0.97 mm, *P* < 0.001). The differences in CRAE and CRVE between boys and girls were not significant. For the adolescents, boys had significant higher MABP (80.2 ± 9.05 mmHg vs. 77.9 ± 9.33, *P* = 0.005) and longer axial length (24.5 ± 1.23 mm vs. 24.1 ± 1.15, *P* < 0.001), but lower levels of blood TC (3.76 ± 0.66 mmol/L vs. 3.98 ± 0.74 mmol/L, *P* < 0.001), HDL-C (1.39 ± 0.29 mmol/L vs. 1.45 ± 0.28 mmol/L, *P* = 0.016), and LDL-C (2.20 ± 0.60 mmol/L vs. 2.33 ± 0.64 mmol/L, *P* = 0.014). Furthermore, boys also had both smaller CRAE (145.5 ± 13.4 μm vs. 150.8 ± 12.8 μm, *P* < 0.001) and smaller CRVE (213.8 ± 20.4 μm vs. 217.8 ± 19.5 μm, *P* = 0.026).

In the younger age group (7–11 y, Table 2), no significant association was found between serum lipid measures and retinal vascular calibres (all *P* > 0.05) after adjusting age, sex, axial length, BMI, MABP and the fellow vascular diameter (i.e., CRVE for CRAE outcomes and *vice versa*). However, in the older age group (12–19 y, Table 3), there was significant retinal arteriolar narrowing with the increases in TG, TC, LDL-C, and apoB (*β*_TG_ = −1.33, *P* = 0.031; *β*_TC_ = −1.83, *P* = 0.003; *β*_LDL-C_ = −1.92, *P* = 0.006; and *β*_apoB_ = −7.18, *P* = 0.009, respectively) after adjusting for age, sex, axial length, BMI, MABP, and CRVE. However, none of the serum lipid parameters was related to retinal venular diameter in adolescents, which was similar to that in the children. When Bonferroni correction was applied (*P*-value threshold = 0.007), TC and LDL-C remain significantly associated with retinal arteriolar narrowing. The association between TG, apoB and CRAE, however, did not show statistical significance.

Table 4 showed the association of retinal arteriolar calibre and dyslipidaemia according to the NHLBI cut-points for plasma lipid, lipoprotein, and apolipoprotein levels in adolescents. For individuals older than 12 years old, abnormalities in TG, TC, LDL-C, and apoB were all significantly associated with narrowing of arteriolar diameter (*P*_*trend*_ = 0.016, 0.042, 0.033 and 0.002, respectively) after adjusting for age, sex, axial length, BMI, MABP and CRVE.

## Discussion

To the best of our knowledge, this is the first study to analyse the relationship between serum lipid profiles, childhood dyslipidaemia and microvascular changes in youth. Using the quantified retinal vascular diameters from digital images, we found no significant association between retinal vascular calibres (both CRAE and CRVE) and any serum lipid indices in children younger than 12 years. However, among adolescents 12 years and older, higher levels of TG, TC, LDL-C, and apoB were significantly associated with narrower retinal arterioles. However, there was no significant association between any lipid parameters and retinal venular calibre in adolescents.

Dyslipidaemia is a strong predictor of developing macrovascular diseases in adults. However, several studies demonstrated that dyslipidaemia might affect microcirculation as well. For instance, higher baseline levels of serum lipids (triglycerides and LDL-C) were associated with greater risk of developing diabetic macular edema and visual impairment in the Early Treatment Diabetic Retinopathy Study (ETDRS)^14^. In a case-control study across 13 countries, higher level of plasma TG and lower level of HDL-C were independently associated with diabetic nephropathy among patients with type 2 diabetes^15^. Based on existing evidence, dyslipidaemia might exert adverse influence on not only macrovascular but also microvascular systems.

Retinal vascular system has been regarded as a classic surrogate of systemic microcirculation. Quantitative analysis for retinal vascular calibre using digital photographs has enabled researchers to investigate the effect of systemic, environmental and genetic factors on microcirculatory network^16^. Several large-scale studies have proved its value in predicting the development of multiple systemic diseases, including hypertension^17^, stroke^18^, coronary heart disease^19^ and diabetes^20^. Although these chronic systemic diseases seldom occurred in children and adolescents, researchers have established the link between adverse retinal vascular changes (i.e. arteriolar narrowing and/or venular widening) and traditional cardio-metabolic risk factors (e.g. obesity^9^ and higher blood pressure^21^). In this study, we observed an association between elevated serum lipids and narrower arterioles, independent of BMI and blood pressure. These findings were to some extent consistent with previous studies, and further validated the association between metabolic abnormalities and microcirculation alterations.

The clinical significance of retinal microvascular changes has not been fully understood yet. Narrowing of retinal arterioles might represent the constriction of systemic arterial vessels, which could cause the increase in peripheral vascular resistance, and eventually led to several systemic conditions. To date, retinal arteriolar narrowing has been documented as an independent predictor for the development of systemic hypertension^22^, diabetes^20^, stroke^23^, chronic kidney disease^24^, metabolic syndrome^25^ and coronary heart disease^26^. Specific to youngsters, among young T1DM patients, smaller arteriolar diameter could independently predict the 16-year development of nephropathy, neuropathy, and proliferative retinopathy^27^. Moreover, retinal vascular geometry parameters, including length-to-diameter ratio, simple tortuosity and fractal dimension, have been reported to precede the incident retinopathy^28^,^29^,^30^ and nephropathy^28^,^31^ in young T1DM individuals. Taken together, these data suggest that retinal vessel signs in early life might precede the onset of systemic microvascular diseases, and could serve as their preclinical markers. Data in our present study also revealed that dyslipidaemia in people aged 12 to 19 years was related to retinal arteriolar narrowing. We therefore speculate that childhood dyslipidaemia may exert an adverse impact on the microvascular system through constricting microvascular arterioles.

Existing data on the relation between lipid profiles and retinal vascular calibres was scarce and inconsistent. Epidemiological surveys, like the Atherosclerosis Risk in Communities Study^32^ and the Cardiovascular Health Study^33^, introduced the same technique as our study to quantify retinal vessel diameters, and found no association of total cholesterol levels with either arteriolar narrowing or AV nicking in population-based adult samples aged 49 years and older. These findings were subsequently confirmed by the Blue Mountain Eye Study^12^. In terms of juveniles, a study among 578 German children aged 10 to 13 years showed that neither serum levels of HDL-cholesterol nor triglycerides were associated with retinal vessel diameters^34^, which was in consistent with our results among children under 12 years old. Moreover, in a health screening program among Japanese adults, persons with elevated TG had significantly narrower retinal arterial diameters^35^, which agreed with our findings in adolescents. Interestingly, our results were well supported by data from clinical trials on cholesterol lowing agents. After LDL apheresis treatment for patients with hypercholesterolemia, the diameters of both retinal arterioles and venules statistically increased^13^. Similarly, in the Age-Related Maculopathy Statin Study, retinal arteriolar caliber of simvastatin group was significantly increased over a 3-year follow-up, while the changes in retinal venular caliber was not significant^36^. Taken together, these evidence indicates an improvement of microvascular perfusion after lipid-lowering intervention.

The patterns of association between serum lipids and retinal vascular calibres may not necessarily be identical across ages. In this study, several lipid parameters were related to CRAE in adolescents, whereas the associations were not statistically significant in children. Similar findings were also revealed by our previous analysis on the association between body composition and retinal vascular calibres^10^. The exact mechanism under the findings may not be readily available, but it might be due to the rapid changes and physical growth across age groups, especially entering into puberty.

The mechanism under the association between the increased atherogenic lipids and retinal arteriolar narrowing in adolescents is potentially complex. One explanation is that elevated plasma lipoproteins could affect endothelial function in both the long and short term^37^,^38^. Endothelial dysfunction could then lead to endothelium-dependent vasoconstriction through inhibiting the L-arginine/NO pathway^39^ and activating the renin-angiotensin system^40^.

There are several limitations that we need to mention. First, as a cross-sectional study, we cannot establish the causal relationship between dyslipidaemia and retinal microvascular changes. Second, blood was drawn under a non-fasting condition and may therefore affect the TG measurement. Fasting time showed little association with lipid subtype levels in both adults and children^41^,^42^, but certain subclass values, especially triglycerides, may differ depending on the fasting status. So there might be a systemic error in triglyceride measurement within our sample due to the fasting time of participants. This might, therefore, contribute to the high proportion of participants with abnormal triglycerides in this study. Third, the LDL-C value was calculated according to Friedewald equation. Although it provides a favorable estimation in most circumstances, errors may be introduced in extreme cases, such as obese children. Fourth, selective bias may exist since the participants were from a twin population. However, the current study sample of twins has been proved to be comparable to population-based singleton samples^43^. More research in general population may be warranted to validate our findings.

In conclusion, we here reported significant associations between several serum lipid parameters, dyslipidaemia and retinal vascular calibres in children and adolescents. This study provided direct evidence that dyslipidaemia could exert adverse impacts on microvascular system from adolescence on. Whether control or normalization of childhood serum lipids would actually leads to beneficial changes in microvasculature remains to be elucidated in long-term longitudinal studies.

## Methods

### Study population

The Guangzhou Twin Registry is an ongoing population-based study launched in 2006 with annual follow up visits^44^,^45^. Approximate 9,700 twin pairs born between 1987 and 2000 were identified through an official household registry and confirmed by door-to-door visit. During the annual visit in July 2009, 2567 participants aged 7 to 19 years, and 2079 (81.0%) accepted retinal photography and retinal vascular calibres measurement. Blood was drawn for serum lipid profiles under a non-fasting condition. Owing to the high correlation of most data between the first- and second-born twin, the first-born twins were arbitrarily selected for analysis to ensure sample independence. We excluded 2 twin pairs with cerebral palsy and 6 with ocular abnormalities (3 with retinopathy of prematurity and 3 with congenital cataract). Individuals with missing data (102 without serum lipid data and 226 without retinal images) were excluded as well, leaving 950 participants in our present study. Due to the significant interaction between age group (age <12 years vs. age >=12 years) and sex on the retinal vessel diameters, we further categorized all participants under the age of 12 years as children, and those of 12 years and older as adolescent for analysis.

This study adhered to the tenets of the Declaration of Helsinki. All procedures were approved by the Ethic Committee of the Zhongshan Ophthalmic Center. Written informed consent was obtained from the parents or legal guardians of all participants under the assent from the children themselves.

### Retinal photography and assessment of retinal vascular calibre

In July 2009, all children underwent retinal photography after pharmacological pupil dilation with 1% cyclopentolate. Retinal images centered at the optic disc were taken by trained nurses using a single fundus camera (Nonmyd 7 digital fundus camera, Kowa, Tokyo, Japan).

We used documented methods to evaluate retinal vascular calibres from digital retinal photographs^32^. All the retinal arterioles and venules within the zone 0.5- to 1-disc diameter (DD) from the optic disc margin were assessed with the computer-assisted software (IVAN, University of Wisconsin, Madison, WI). Trained graders analyzed retinal images according to a standardized protocol provided by the Retinal Vascular Imaging Centre (University of Melbourne, Australia). General information and clinical data of participants were blinded to image graders. The average diameters of retinal arteriolar and venular were summarized as central retinal arteriolar equivalent (CRAE) and venular equivalent (CRVE) to the nearest 0.1 micron, respectively^46^. To control the quality, 50 retinal images were randomly selected and re-evaluated by the same grader with a 4-week interval, providing intra-class correlation (ICC) coefficients >0.90 for both CRAE and CRVE.

### Laboratory analyses

Blood sampling was drawn under a non-fasting status. Samples were taken by venipuncture of an antecubital vein using vacuum tubes in a sitting position. Blood samples were collected by qualified medical personnel and immediately transported to the laboratory for further analysis. The following serum lipid parameters were analyzed using standardized procedures according to the manufacturer’s recommendations. Total cholesterol (TC), triglycerides (TG) and high-density lipoprotein cholesterol (HDL-C) were determined directly, and low-density lipoprotein cholesterol (LDL-C) was calculated based on the Friedewald equation^47^.

### Other Measurements

Blood pressure (BP) was measured in the sitting position after a five-minute rest. Three separate measurements were taken to generate the mean for analysis. Mean arterial blood pressure (MABP) was calculated as one third of the systolic BP (SBP) plus two thirds of the diastolic BP (DBP). Axial length of the eyeball was obtained from a laser interferometer (IOLMaster, Carl Zeiss). Body weight was measured to the nearest 0.1 kg, and height to the nearest 0.1 cm. Body mass index (BMI) was computed as weight in kilograms divided by the square of height in meters.

### Statistical analysis

Due to the high correlation between the two eyes in each individual, only data from the right eyes were arbitrary chosen for analysis. In multivariable regression models, we introduced CRAE or CRVE as dependent variables and assessed their association with each lipid parameters: TG, TC, HDL-C, LDL-C, apoA1, apoB, and apoA1-to-apoB ratio (apoA1/apoB). Covariates including age, sex, MABP, axial length and the fellow vascular diameter (i.e., CRVE for CRAE outcomes and *vice versa*)^48^,^49^ were adjusted. Based on the cutoff points set by the NHLBI in 2011^6^, we divided the participants into subgroups (i.e. acceptable, borderline and abnormal) and explored the association of dyslipidaemia and retinal vascular calibres. For statistical significance in all testing, we set the *P*-value threshold at 0.05 unless otherwise specified. Considering the increasing chance of committing type I error in multiple comparisons, we also set *P*-value threshold to be 0.007 according to Bonferroni correction for simultaneously testing the significance of the 7 predictors in the multivariate analyses. Statistical analyses were conducted by using STATA software (version 13.0, StataCorp LP, TX, USA).

## Additional Information

**How to cite this article:** Xiao, W. *et al*. Serum lipid profiles and dyslipidaemia are associated with retinal microvascular changes in children and adolescents. *Sci. Rep.* **7**, 44874; doi: 10.1038/srep44874 (2017).

**Publisher's note:** Springer Nature remains neutral with regard to jurisdictional claims in published maps and institutional affiliations.

## Figures and Tables

**Table 1:** Characteristics of enrolled participants.

Variables	Younger group (<12-year old, n = 438)			Older group (≥12-year old, n = 512)		
	Boys (n = 214)	Girls (n = 224)	*P*	Boys (n = 239)	Girls (n = 273)	*P*
Sex, %	48.9	51.1	N/A*	46.7	53.3	N/A*
Age, year	9.79 ± 1.46	9.69 ± 1.43	0.447	14.8 ± 1.98	15.0 ± 1.94	0.281
SBP, mmHg	95.6 ± 13.2	94.1 ± 12.0	0.210	113.1 ± 12.9	106.0 ± 12.1	<0.001
DBP, mmHg	57.6 ± 10.4	57.8 ± 8.93	0.864	63.7 ± 9.16	63.8 ± 9.52	0.899
MABP, mmHg	70.3 ± 10.6	69.9 ± 9.36	0.676	80.2 ± 9.05	77.9 ± 9.33	0.005
BMI, kg/m^2^	16.7 ± 2.80	16.4 ± 2.64	0.233	19.4 ± 3.25	19.1 ± 2.75	0.225
TG, mmol/L	1.35 ± 0.69	1.41 ± 0.75	0.389	1.39 ± 0.68	1.36 ± 0.75	0.713
TC, mmol/L	3.94 ± 0.74	4.03 ± 0.72	0.191	3.76 ± 0.66	3.98 ± 0.74	<0.001
HDL-C, mmol/L	1.44 ± 0.31	1.41 ± 0.30	0.328	1.39 ± 0.29	1.45 ± 0.28	0.016
LDL-C, mmol/L	2.22 ± 0.61	2.36 ± 0.59	0.022	2.20 ± 0.60	2.33 ± 0.64	0.014
apoA1, g/L	1.43 ± 0.21	1.38 ± 0.18	0.021	1.33 ± 0.17	1.36 ± 0.18	0.107
apoB, g/L	0.75 ± 0.15	0.76 ± 0.16	0.288	0.78 ± 0.15	0.80 ± 0.17	0.075
apoA1/apoB	1.98 ± 0.49	1.88 ± 0.42	0.017	1.77 ± 0.38	1.77 ± 0.44	0.917
Axial length, mm	23.7 ± 1.01	23.2 ± 0.97	< 0.001	24.5 ± 1.23	24.1 ± 1.15	<0.001
CRAE, μm	151.0 ± 13.1	153.1 ± 12.2	0.074	145.5 ± 13.4	150.8 ± 12.8	<0.001
CRVE, μm	219.0 ± 17.7	221.5 ± 17.0	0.131	213.8 ± 20.4	217.8 ± 19.5	0.026

Data are mean ± SD or proportions. *N/A, not applicable.

**Table 2:** Association of retinal vascular calibers and lipid profiles in children (<12-year-old).

Serum lipid parameters	Central retinal arteriolar caliber				Central retinal venular caliber			
	Regression model 1		Regression model 2		Regression model 1		Regression model 2	
	β (95% CI)	*P*	β (95% CI)	*P*	β (95% CI)	*P*	β (95% CI)	*P*
TG, mmol/L	−0.03 (−1.66, 1.61)	0.976	0.37 (−1.08, 1.81)	0.620	1.16 (−1.10, 3.42)	0.315	1.14 (−0.80, 3.09)	0.248
TC, mmol/L	1.63 (−0.01, 3.26)	0.051	1.18 (−0.23, 2.58)	0.101	2.00 (−0.27, 4.26)	0.084	0.43 (−1.48, 2.33)	0.660
HDL-C, mmol/L	−1.02 (−4.90, 2.86)	0.606	−1.19 (−4.58, 2.18)	0.487	−2.08 (−7.46, 3.30)	0.447	−0.94 (−5.49, 3.61)	0.685
LDL-C, mmol/L	1.93 (−0.03, 3.90)	0.054	1.49 (−0.21, 3.20)	0.085	2.75 (0.02, 5.47)	0.048	1.05 (−1.26, 3.35)	0.373
apoA1, g/L	1.15 (−4.84, 7.15)	0.706	1.30 (−3.81, 6.40)	0.618	−3.74 (−12.0, 4.56)	0.376	−5.30 (−12.1, 1.54)	0.128
apoB, g/L	5.03 (−2.51, 12.6)	0.190	4.85 (−1.67, 11.4)	0.144	5.52 (−4.93, 15.9)	0.300	0.26 (−8.55, 9.07)	0.954
apoA1/apoB	−1.43 (−3.98, 1.13)	0.272	−1.44 (−3.64, 0.76)	0.199	−1.96 (−5.50, 1.58)	0.276	−0.80 (−3.77, 2.17)	0.597

Variables adjusted in multiple regression model 1: age and sex.

Variables adjusted in multiple regression model 2: age, sex, axial length, BMI, MABP and the fellow retinal vascular caliber.

**Table 3:** Association of retinal vascular calibres and lipid profiles in adolescents (≥12-year-old).

Serum lipid parameters	Central retinal arteriolar calibre				Central retinal venular calibre			
	Regression model 1		Regression model 2		Regression model 1		Regression model 2	
	β (95% CI)	*P*	β (95% CI)	*P*	β (95% CI)	*P*	β (95% CI)	*P*
TG, mmol/L	−1.54 (−3.11, 0.03)	0.055	−1.33 (−2.54, −0.12)	0.031	0.46 (−1.95, 2.86)	0.709	0.90 (−0.94, 2.75)	0.335
TC, mmol/L	−1.13 (−2.73, 0.47)	0.165	−1.83 (−3.04, −0.62)	0.003	0.89 (−1.54, 3.33)	0.473	0.65 (−1.21, 2.51)	0.494
HDL-C, mmol/L	−1.96 (−5.87, 1.94)	0.324	−1.20 (−4.26, 1.86)	0.442	−5.47 (−11.4, 0.49)	0.072	−1.73 (−6.34, 2.89)	0.463
LDL-C, mmol/L	−1.17 (−2.98, 0.63)	0.200	−1.92 (−3.30, −0.55)	0.006	1.26 (−1.49, 4.01)	0.367	0.68 (−1.43, 2.79)	0.527
apoA1, g/L	−6.13 (−12.5, 0.19)	0.057	−4.63 (−9.49, 0.23)	0.062	−5.85 (−15.5, 3.82)	0.235	1.43 (−5.96, 8.82)	0.704
apoB, g/L	−6.27 (−13.3, 0.78)	0.081	−7.18 (−12.6, −1.78)	0.009	1.08 (−9.71, 11.9)	0.844	0.56 (−7.71, 8.83)	0.895
apoA1/apoB	0.15 (−2.58, 2.88)	0.915	1.15 (−0.96, 3.26)	0.284	−2.49 (−6.65, 1.67)	0.240	−0.12 (−3.32, 3.08)	0.943

Variables adjusted in multiple regression model 1: age and sex.

Variables adjusted in multiple regression model 2: age, sex, axial length, BMI, MABP and the fellow retinal vascular caliber.

**Table 4:** Association of retinal arteriolar caliber and dyslipidaemia in adolescents (≥12-year-old).

Dyslipidemia	N (%)	Crude CRAE	Multiple regression model
TG, mmol/L			
Acceptable	180 (35.2)	149.8 ± 13.7	149.9 ± 9.15
Borderline high	171 (33.4)	147.8 ± 13.2	147.8 ± 9.01
Abnormal	161 (31.4)	147.3 ± 13.0	147.2 ± 9.27
*P* for trend		0.179	0.016
TC, mmol/L			
Acceptable	409 (79.9)	148.8 ± 13.6	148.8 ± 9.58
Borderline high	79 (15.4)	147.1 ± 12.6	147.1 ± 7.89
Abnormal	24 (4.7)	144.6 ± 10.3	144.6 ± 5.48
*P* for trend		0.225	0.042
LDL-C, mmol/L			
Acceptable	428 (83.6)	148.6 ± 13.6	148.6 ± 9.48
Borderline high	62 (12.1)	148.2 ± 12.0	148.2 ± 7.91
Abnormal	22 (4.3)	143.4 ± 11.2	143.4 ± 5.76
*P* for trend		0.198	0.033
ApoB, g/L			
Acceptable	404 (78.9)	148.9 ± 13.7	148.9 ± 9.39
Borderline high	91 (17.8)	145.4 ± 11.0	145.4 ± 7.98
Abnormal	17 (3.3)	141.2 ± 14.6	141.2 ± 7.17
*P* for trend		0.050	0.002

Variables adjusted in multiple regression model: age, sex, axial length, BMI, MABP and CRVE.
